# Supplementary material for: Translation, Cross-Cultural Adaptation and Psychometric Validation of the Greek Version of the Cardiac Rehabilitation Barriers Scale (CRBS-GR): What Are the Barriers in South-East Europe?
Source: Int J Environ Res Public Health. 2023 Feb 24;20(5):4064. doi: 10.3390/ijerph20054064 (PMC10002300; doi:10.3390/ijerph20054064)
Supplement: Supplementary file 1 [file ijerph-20-04064-s001.zip › ijerph-2176088-supplementary.pdf]

Supplementary Table S1. CRBS-GR version

Κλίμακα Εμποδίων Καρδιακής Αποκατάστασης

Οι παρακάτω ερωτήσεις αφορούν ορισμένους από τους παράγοντες που επηρεάζουν τη συμμετοχή σας σε συνεδρίες καρδιακής αποκατάστασης. Απαντήστε σε **όλες τις ερωτήσεις** σε αυτήν τη σελίδα, ανεξάρτητα από το εάν παρακολουθήσατε ή όχι πρόγραμμα καρδιακής αποκατάστασης.

Δεν παρακολούθησα πρόγραμμα καρδιακής αποκατάστασης ή αν παρευρέθηκα, έχασα κάποιες συνεδρίες εξαιτίας των παρακάτω:

	Διαφωνώ απόλυτα	Διαφωνώ	Ούτε συμφωνώ Ούτε διαφωνώ	Συμφωνώ	Συμφωνώ απόλυτα
1. … της απόστασης (π.χ. δεν βρίσκεται στην περιοχή σας, πολύ μακριά για να ταξιδέψετε)	☐	☐	☐	☐	☐
2. …του κόστους (π.χ. παρκινγκ, καύσιμα)	☐	☐	☐	☐	☐
3. …δυσκολίας μετακίνησης (π.χ. πρόσβαση σε αυτοκίνητο ,Μέσα Μαζικής Μεταφοράς)	☐	☐	☐	☐	☐
4. …οικογενειακών υποχρεώσεων	☐	☐	☐	☐	☐
5. …έλλειψη ενημέρωσης σχετικά με καρδιακή αποκατάσταση (π.χ. δεν είχα ενημέρωση από το γιατρό μου)	☐	☐	☐	☐	☐
6. …δε χρειάζομαι καρδιακή αποκατάσταση	☐	☐	☐	☐	☐
7. …ασκούμαι ήδη στο σπίτι ή στην κοινότητα μου	☐	☐	☐	☐	☐
8. …άσχημων καιρικών συνθηκών	☐	☐	☐	☐	☐
9. …θεωρώ την άσκηση κουραστική ή επώδυνη	☐	☐	☐	☐	☐

10. …συχνά ταξίδια (π.χ. διακοπές, επαγγελματικά, εξοχική κατοικία) ☐ ☐ ☐ ☐ ☐

11. …των χρονικών περιορισμών (π.χ. πολύ απασχολημένος) ☐ ☐ ☐ ☐ ☐

12. …εργασιακές υποχρεώσεις ☐ ☐ ☐ ☐ ☐

13. …έλλειψης ενέργειας ☐ ☐ ☐ ☐ ☐

14. …άλλων θεμάτων υγείας με εμποδίζουν να συμμετέχω (προσδιορίστε:______) ☐ ☐ ☐ ☐ ☐

15. …ηλικία (μεγάλος) ☐ ☐ ☐ ☐ ☐

16. …δεν το θεώρησε ο γιατρός μου απαραίτητο ☐ ☐ ☐ ☐ ☐

17. … πολλοί άνθρωποι με καρδιακά προβλήματα δεν πάνε και είναι καλά ☐ ☐ ☐ ☐ ☐

18. … μπορώ να διαχειριστώ μονός μου το πρόβλημα της καρδιάς μου ☐ ☐ ☐ ☐ ☐

19. … νομίζω ότι παραπέμφθηκα αλλά το πρόγραμμα δεν επικοινώνησε μαζί μου ☐ ☐ ☐ ☐ ☐

20. …πήρε πολύ χρόνο για να ενημερωθώ και να μπω στο πρόγραμμα ☐ ☐ ☐ ☐ ☐

21. …προτιμώ να φροντίζω μόνος/η μου την υγεία μου και όχι μέσα από τη συμμετοχή μου σε ομάδες ☐ ☐ ☐ ☐ ☐

Supplementary Table S2. Results for the -3 weeks interval-reproducibility test by subscale

Subscale	ICC	95% CI

Comorbidities/ Functional status	0.97	[0.92 – 0.99]
Logistical factors	0.97	[0.92 – 0.99]
Work constraints/ Lack of time	0.91	[0.77 – 0.97]
Perceived need/Healthcare factors	0.86	[0.64 – 0.95]
Total	0.96	[0.90 – 0.99]

ICC, intra-class correlation; CI, confidence interval

Supplementary Table S3. Results of the Chi-square and One-Way ANOVA tests between the barriers and the characteristics of the participants.

Barriers	Gender		Distance		Education		BMI		Hyperlipidemia		Tobacco use		Age	
	X^2	*p-value*	X^2	*p-value*	X^2	*p-value*	X^2	*p-value*	X^2	*p-value*	X^2	*p-value*	*F*	*p-value*
1. Distance (e.g., it is not located inyour area, too far to travel)	4.96	0.291	49.8	0.000	12.7	0.124	15.1	0.236	4.62	0.329	7.45	0.114	4.11	0.004
2. Costs (e.g., parking, fuel)	8.16	0.086	25.0	0.000	16.3	0.038	8.88	0.713	8.08	0.089	4.48	0.344	1.59	0.182
3. Mobility difficulties (e.g.,	11.74	0.019	11.1	0.025	12.2	0.144	12.7	0.390	0.37	0.985	2.86	0.582	2.20	0.074

accessin a car, Public Transport)														
4. Family obligations	9.17	0.057	0.27	0.991	3.44	0.903	10.1	0.603	2.46	0.651	1.76	0.781	1.52	0.201
5. Lack of information about cardiac rehabilitation (e.g., I did not have an update from my doctor)	4.87	0.301	3.62	0.461	9.24	0.322	12.7	0.389	7.35	0.119	2.96	0.564	0.12	0.977
6. I don't need cardiac rehabilitation	1.65	0.800	3.04	0.552	2.43	0.965	9.44	0.665	11.8	0.019	4.40	0.355	2.94	0.024
7. I already practice at home or in my community	4.64	0.326	9.18	0.057	11.4	0.182	12.8	0.381	6.56	0.161	4.64	0.326	0.71	0.588
8. Bad weather conditions	8.69	0.069	7.56	0.109	4.71	0.788	13.1	0.361	4.26	0.372	1.83	0.767	0.42	0.797
9. I find exercise tiring or painful	4.72	0.318	3.93	0.416	20.7	0.008	6.78	0.872	2.34	0.674	2.48	0.648	2.63	0.039
10. Frequent trips (e.g., holidays,business, holiday home)	3.00	0.558	1.11	0.893	8.72	0.367	8.47	0.748	1.24	0.872	6.76	0.149	2.84	0.028
11. Time constraints (e.g., very busy)	0.49	0.974	1.06	0.900	7.81	0.452	18.7	0.097	4.65	0.326	3.21	0.523	2.43	0.053
12. Work commitments	4.78	0.311	1.84	0.765	8.43	0.392	17.7	0.125	3.33	0.505	8.74	0.068	15.4	0.000
13. Lack of energy	7.24	0.124	7.66	0.105	16.4	0.038	4.81	0.964	1.42	0.841	2.63	0.622	0.65	0.629
14. Other health issues that prevent me fromparticipating (specify:___)	2.47	0.650	1.02	0.907	11.0	0.203	12.5	0.409	2.46	0.651	3.94	0.414	0.49	0.741
15. Age (old)	1.90	0.754	3.97	0.411	12.5	0.132	11.2	0.512	0.60	0.963	4.86	0.302	7.85	0.000

16. My doctor didn't think it was so essential	5.53	0.237	1.11	0.893	6.82	0.557	16.9	0.153	1.85	0.763	6.18	0.186	0.91	0.463
17. Many people with cardiacproblems do not go and they are fine	5.14	0.273	3.81	0.433	3.05	0.931	6.91	0.863	0.83	0.935	5.40	0.249	0.43	0.789
18. I can handle my heart problem on my own	2.16	0.706	0.98	0.913	8.79	0.361	13.1	0.364	3.88	0.422	2.27	0.686	2.06	0.091
19. I think I got referred but the program did not contact me	3.58	0.466	3.09	0.543	6.30	0.614	8.80	0.721	7.70	0.103	5.60	0.231	1.80	0.134
20. It took me a long time to catch up and to enter the program	2.59	0.629	4.56	0.335	19.9	0.011	10.5	0.573	2.52	0.641	7.77	0.101	2.64	0.038
21. I prefer to take care of my health on my own and not through participating in groups	5.12	0.275	4.25	0.373	9.23	0.323	8.58	0.739	3.34	0.503	1.02	0.907	1.60	0.180

BMI, body mass index; ANOVA, analysis of variance

Supplementary Table S4. Results of the concurrent validity test between the four factors and the total scores of the patients' responses in the HADS Questionnaire.

Subscale	Correlation
Comorbidities/ Functional status	0.56
Logistical factors	0.47
Work constraints/ Lack of time	0.46
Perceived need/Healthcare factors	0.50

HADS, Hospital Depression and Anxiety Scale
